# Five years of post-marketing liver safety data from the tolvaptan Risk Evaluation and Mitigation Strategy

**DOI:** 10.1093/ckj/sfaf062

**Published:** 2025-02-25

**Authors:** Emanuel Lohrmann, Thomas Jaeger, Kene Enekebe, Zhen Zhang, Timothy Wilt, Michele Riggen, Annette Stemhagen, Indu Nair, Sasikiran Nunna, Ancilla W Fernandes, Hema Gandhi, Olga Sergeyeva, Vinu George

**Affiliations:** Otsuka Pharmaceutical GmbH, Frankfurt, Germany; Otsuka Pharmaceutical GmbH, Frankfurt, Germany; Otsuka Pharmaceutical Development & Commercialization Inc., Princeton, NJ, USA; Otsuka Pharmaceutical Development & Commercialization Inc., Princeton, NJ, USA; Otsuka Pharmaceutical Development & Commercialization Inc., Rockville, MD, USA; UBC, Blue Bell, PA, USA; UBC, Blue Bell, PA, USA; UBC, Blue Bell, PA, USA; Otsuka Pharmaceutical Development & Commercialization Inc., Princeton, NJ, USA; Otsuka Pharmaceutical Development & Commercialization Inc., Rockville, MD, USA; Otsuka Pharmaceutical Development & Commercialization Inc., Princeton, NJ, USA; Otsuka Pharmaceutical Development & Commercialization Inc., Rockville, MD, USA; Otsuka Pharmaceutical Development & Commercialization Inc., Rockville, MD, USA

**Keywords:** autosomal dominant polycystic kidney disease (ADPKD), drug-induced liver injury (DILI), liver safety, post-marketing surveillance, tolvaptan

## Abstract

**Background:**

Approval of tolvaptan in the USA for the treatment of autosomal dominant polycystic kidney disease (ADPKD) was contingent on implementation of a Risk Evaluation and Mitigation Strategy (REMS) that includes monitoring for drug-induced liver injury (DILI). Liver safety data from the REMS were published previously for the period from program start (May 2018) to February 2021. To further characterize the post-marketing liver safety of tolvaptan, we provide a REMS update.

**Methods:**

We analyzed prospective pharmacovigilance data on ADPKD patients who initiated tolvaptan in the post-marketing setting. The data capture period was May 2018 to February 2023.

**Results:**

Among 10 879 tolvaptan-treated patients, exposure was >12 months for 45% and >18 months for 35%. Since the 3-year analysis, in which 60/6711 (0.9%) patients were reported with possible severe DILI, the frequency has remained consistent for 5 years [i.e. 82/10 879 (0.8%)]. Incidence of possible severe DILI in the REMS at 5 years was 0.52 events per 100 patient years. Confirmation of possible severe DILI events as serious and potentially fatal was made for 4/82 events, with no new cases confirmed since the 3-year reporting period. No fatalities or liver transplants attributable to tolvaptan-related DILI have been reported in the REMS.

**Conclusions:**

Five years of data support adherence to the per-label tolvaptan liver function monitoring schedule to promptly detect and manage liver toxicity. Conclusions are limited by data availability at the time of analysis cutoff, with the possibility that additional cases of possible severe DILI and/or serious and potentially fatal events may be identified.

## Graphical Abstract

**Graphical Abstract:**
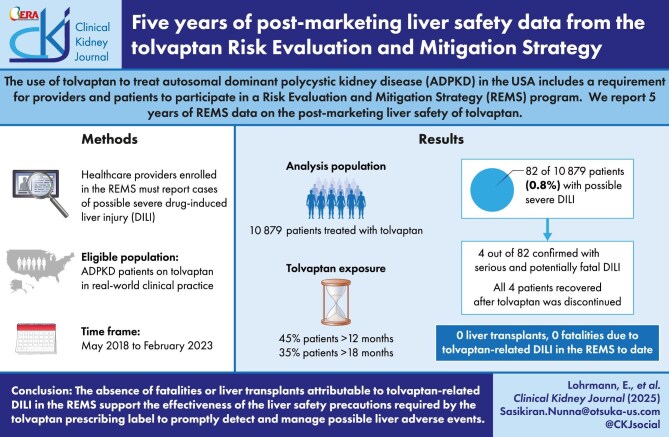

KEY LEARNING POINTS **What was known:**

- In clinical trials of tolvaptan for the treatment of autosomal dominant polycystic kidney disease (ADPKD), a liver safety signal was detected.
- Regulatory approval in the USA was contingent on post-marketing liver safety surveillance conducted as part of a Risk Evaluation and Mitigation Strategy (REMS).
- An interim analysis of 3-year REMS data for >6000 patients supported the value of adhering to the per-label liver function testing schedule to mitigate risk of adverse outcomes, but more data are needed to better characterize the tolvaptan liver safety profile.

**This study adds:**

- This updated analysis of liver safety data from the tolvaptan REMS evaluated >10 000 patients over a 5-year period (May 2018–February 2023).
- Compared to the earlier 3-year analysis, in which 0.9% of patients were reported to have possible severe drug-induced liver injury (DILI) events, the 5-year data were similar (0.8% of patients).
- No new cases of serious or potentially fatal DILI were confirmed since the previous reporting period, and no fatalities or liver transplants attributable to tolvaptan-related DILI have been reported in the REMS overall.

**Potential impact:**

- The lack of fatalities or transplants attributable to tolvaptan-related DILI in the REMS at time of data cutoff indicates that the required regular liver function monitoring with tolvaptan use enables prompt detection of liver abnormalities and appropriate management to reduce risk of adverse outcomes.
- Extensive post-marketing data from the REMS increase the confidence that adherence to per-label liver function monitoring is effective in mitigating risk of hepatotoxicity in ADPKD patients receiving tolvaptan.
- Conclusions are limited, given that the data available are restricted to the reported analysis period. Continued adherence to prescribing information requirements regarding liver safety monitoring and ongoing pharmacovigilance are needed.

## INTRODUCTION

A liver safety signal was detected in the TEMPO 3:4 clinical trial of tolvaptan for the treatment of patients with autosomal dominant polycystic kidney disease (ADPKD), in which there was an imbalance in the proportion of participants with alanine aminotransferase (ALT) levels more than three times the upper limit of normal (ULN) between the tolvaptan (4.4%) and placebo (1.0%) trial arms [1–3]. Additionally, three cases of Hy's Law [i.e. ALT or aspartate aminotransferase (AST) more than or equal to three times the ULN and total bilirubin more than twice the ULN, in the absence of cholestasis and without any other reason to explain the elevations] occurred in TEMPO 3:4 (two cases) and its extension TEMPO 4:4 (1 case) [2–4]. Liver function monitoring was increased for the REPRISE clinical trial to once monthly. Under the once-monthly schedule, there was a greater proportion of participants in the tolvaptan arm (5.6%) than in the placebo arm (1.2%) of REPRISE who experienced ALT more than three times the ULN, but no additional Hy's Law cases were reported in REPRISE, nor in a long-term, open-label extension, in which testing was conducted monthly for the first 18 months of tolvaptan exposure and once every 3 months thereafter [3, 5, 6].

Regulatory approval of tolvaptan for the treatment of patients with ADPKD who are at increased risk of rapid progression was contingent on commitments to post-marketing pharmacovigilance [7, 8]. Per the US tolvaptan label, liver function testing in tolvaptan-treated patients must be conducted before tolvaptan initiation (baseline), at 2 weeks and 4 weeks after initiation, then continuing monthly for the first 18 months and every 3 months thereafter [9]. Data from safety monitoring of patients treated with tolvaptan for ADPKD in routine practice have been reported. An interim analysis of 3-year data collected from the Risk Evaluation and Mitigation Strategy (REMS) program, which enrolls all US patients receiving tolvaptan for ADPKD, has been published [10]. The reporting period was from REMS initiation (14 May 2018) to 23 February 2021. Among 6711 previously tolvaptan-naïve patients who participated in the REMS, four patients experienced confirmed serious and potentially fatal drug-induced liver injury (DILI), including one who met Hy's Law criteria. All recovered after tolvaptan discontinuation. No fatalities or liver transplants due to tolvaptan use were reported [10]. An update from the REMS has since been provided with interim 4-year data, from program start to 23 February 2022 [11]. No new cases of confirmed serious and potentially fatal DILI were reported, yielding an incidence of 4 out of 8764 cases during 4-year follow-up. Again, no fatalities or liver transplants attributed to tolvaptan were reported, supporting the appropriateness of once-monthly liver enzyme monitoring to enable prompt detection and management of liver function abnormalities to avert more serious injury.

In both interim analyses of the REMS data, the incidence of possible severe DILI was lower than in the clinical development program, although this finding was only preliminary due to the shorter length of follow-up for patients in the REMS compared to clinical trials [10, 11]. To further characterize the liver safety profile of tolvaptan in the post-marketing setting, safety monitoring and reporting continues. Here we report an updated interim analysis of REMS data with ∼5 years of follow-up.

## MATERIALS AND METHODS

### Eligibility

Eligibility criteria for the analysis were: patients with ADPKD who were enrolled in the REMS in the USA, who were tolvaptan-naïve, and who initiated treatment with tolvaptan in the post-marketing setting. The exclusion of patients who had previously received tolvaptan in clinical trials was intended to obtain time-linked safety data that commenced with tolvaptan initiation.

### Study design

The design of this retrospective analysis of US patients enrolled in the tolvaptan REMS was similar to that of the 3-year and 4-year interim REMS analyses [10, 11]. Data sources were the REMS database and the Otsuka Global Pharmacovigilance (GPV) database. The GPV department collects detailed follow-up information from tolvaptan prescribers regarding any reports of possible severe DILI, using an Enhanced Pharmacovigilance Form. The information obtained in this manner is entered into the GPV database.

The reporting period for the present analysis is ∼5 years: 14 May 2018–23 February 2023.

### Identification of possible severe DILI

Adverse events of possible severe DILI were identified using criteria identical to those previously reported [10]. Possible cases were defined as any patient who met at least one of the following three criteria, regardless of the assessment of a possible causal relationship with tolvaptan in the judgment of the reporter:

- Diagnosis of acute hepatic failure, hepatic failure, hepatic necrosis, hepatic encephalopathy, ascites, hepatorenal failure, hepatorenal syndrome, fulminant hepatitis, liver transplant, or death related to liver injury.
- Development of any adverse event matching a lower-level Medical Dictionary for Regulatory Activities (MedDRA) term in one of the five hepatic Standardized MedDRA Queries (SMQs) (Table 1), and leading to liver transplantation, resulting in a fatal outcome, or considered to be life-threatening.
- Development of any adverse event matching a lower-level MedDRA term in one of the five hepatic SMQs and meeting any prespecified liver enzyme elevation as defined by the US Food and Drug Administration to guide treatment discontinuation [12] (Table 1).

**Table 1:** SMQs and laboratory criteria used to identify possible severe DILI.

Hepatic SMQs	Laboratory criteria
• Cholestasis and jaundice of hepatic origin • Hepatic failure, fibrosis and cirrhosis, and other liver damage-related conditions • Hepatitis, noninfectious • Liver-related investigations, signs, and symptoms • Liver-related coagulation and bleeding disturbances	• ALT or AST >8× ULN • ALT or AST >5× ULN for >2 weeks • ALT or AST >3× ULN and total bilirubin >2× ULN or INR >1.5 (total bilirubin measurement can be within 30 days of the ALT elevation) • ALT or AST >3× ULN with the appearance of fatigue, nausea, vomiting, right upper quadrant pain or tenderness, fever, rash, and/or eosinophilia (>5%)

INR, international normalized ratio.

Cases of possible severe DILI were further evaluated to identify events belonging to the following categories:

#### Hy's Law

ALT or AST more than or equal to three times the ULN and total bilirubin more than twice the ULN, in the absence of cholestasis and without any other reason to explain the elevations [12].

#### Confirmed severe DILI

The FDA defines severe liver injury as irreversible liver failure that is fatal or requires transplantation [12].

#### Serious and potentially fatal liver injury

Physicians or specialty pharmacists reporting a liver-related adverse event were asked to indicate whether the event was serious and potentially fatal. Initially, the designation was based solely on the clinical judgment of the reporter. From February 2020, a more detailed Liver Adverse Event Reporting Form and Patient Status form and updated Prescriber Training content were introduced to better capture relevant patient data and provide more precise criteria for healthcare providers to determine whether serious and potentially fatal liver injury occurred [10]. Events reported before and after the introduction of the new reporting and training material require confirmation as serious and potentially fatal by the Otsuka GPV team. In making the determination, the team followed up to obtain relevant evidence as described by FDA guidance, including laboratory data and trends in those data over time, diagnostic tests, symptoms, medical history, the time-course of the event, and information about potential confounding risk factors and concomitant medications [12].

### Analyses

Summary statistics are presented for patient characteristics, tolvaptan exposure, and description of possible severe DILI (time to event, laboratory data, and outcomes). Average daily tolvaptan dose for each subject was calculated as the sum of total daily dose divided by the number of days of supply. Comparison of the incidence of possible severe DILI between the REMS and the tolvaptan clinical trial program was made by calculating the incidence rate ratio (IRR) using the Wald method. The clinical trials included in the comparison were TEMPO 3:4; its extension TEMPO 4:4; REPRISE; and a long-term extension that enrolled participants from REPRISE, TEMPO 4:4, and previous tolvaptan trials [1, 4–6].

### Ethical conduct

The study received an exemption from the central Institutional Review Board (Sterling IRB; Atlanta, GA, USA).

## RESULTS

### Patient disposition

A total of 13 508 patients enrolled in the REMS during the reporting period. Among the total enrolled, 11 655 initiated tolvaptan, 10 879 (93%) of whom were tolvaptan-naïve at the time of enrollment and hence were included in the analysis.

### Demographic and clinical characteristics

The sex distribution of the analysis population was roughly even (Table 2). Around 78% were aged ≤55 years, with a mean age at enrollment of 46 years. As a revised REMS Patient Enrollment Form with more detailed questions was not introduced until February 2019, data on patient race, ethnicity, and alcohol consumption were missing for one-fifth of the patients enrolled (∼19%). Among the patients for whom data was available, most were White (75.7%), not Hispanic or Latino (87.0%), and current alcohol drinkers (53.9%; see Table 2 for more detailed demographic data and alcohol consumption categories).

**Table 2:** Baseline demographic and clinical characteristics.

Variable	Overall analysis population (*N* = 10 879)	Subset with possible severe DILI (*n* = 82)
Age (years) at enrollment		
Mean (SD)	45.5 (12.42)	47.1 (11.80)
Range	14, 95	18, 68
Sex, *n* (%)		
Female	5611 (51.6)	52 (63.4)
Male	5268 (48.4)	30 (36.6)
Age, *n* (%)		
≤55 Years	8503 (78.2)	61 (74.4)
>55 Years	2376 (21.8)	21 (25.6)
Race, *n* (%)		
Race unknown	2078 (19.1)	44 (53.7)
Race known^a^	8801 (80.9)	38 (46.3)
White	6660 (75.7)	22 (57.9)
Black or African American	989 (11.2)	8 (21.1)
American Indian or Alaska Native	46 (0.5)	1 (2.6)
Asian	485 (5.5)	4 (10.5)
Native Hawaiian or Other Pacific Islander	28 (0.3)	0 (0.0)
Other	593 (6.7)	3 (7.9)
Ethnicity		
Ethnicity unknown	2101 (19.3)	44 (53.7)
Ethnicity known^b^	8778 (80.7)	38 (46.3)
Hispanic or Latino	1144 (13.0)	6 (15.8)
Not Hispanic or Latino	7634 (87.0)	32 (84.2)
Alcohol classification, *n* (%)		
Classification unknown	2079 (19.1)	45 (54.9)
Classification known^c^	8800 (80.9)	37 (45.1)
Never drank	3032 (34.5)	19 (51.4)
Ex-drinker (stopped drinking ≥1 month ago)	1023 (11.6)	3 (8.1)
Current drinker	4745 (53.9)	15 (40.5)
Typical alcohol consumption (current drinkers only)^d^		
Occasional (drink alcohol <1 time/week)	3078 (64.9)	9 (60.0)
Light (1–2 drinks/week)	1006 (21.2)	2 (13.3)
Moderate (3–7 drinks/week)	557 (11.7)	4 (26.7)
Heavy (>7 drinks/week)	104 (2.2)	0 (0.0)

a The denominator for the percentage in each racial category shown is the number of patients with race known.

^b^The denominator for the percentage in each ethnic category shown is the number of patients with ethnicity known.

^c^The denominator for the percentage in each alcohol classification shown is the number of patients with classification known.

^d^The denominator for the percentage in each typical alcohol consumption category shown is the number of patients who were current drinkers.

SD, standard deviation.

### Exposure

**Figure 1:**
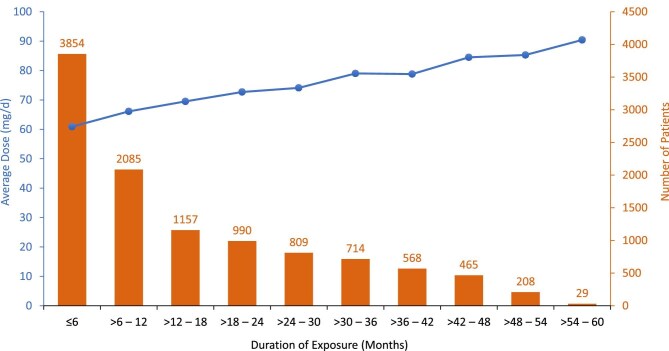
Average tolvaptan dose and number of patients by duration of exposure (analysis population, *N* = 10 879).

Among the analysis population, 45% had taken tolvaptan for >12 months, and 35% had taken tolvaptan for >18 months (Fig. 1). The lower percentages of patients with longer tolvaptan exposures reflected increasing enrollment in the REMS during the analysis period, as more patients started tolvaptan treatment over time, resulting in a greater proportion of the analysis population having shorter exposures. The average daily tolvaptan dose was 60 mg or greater, with average doses higher in patients exposed for longer periods (Fig. 1).

### Cases of possible severe DILI

At the time of the 4-year interim analysis, 69/8764 (0.8%) patients were identified as having possible severe DILI [11]. Since that time, an additional 13 previously tolvaptan-naïve patients in the US REMS met criteria for possible severe DILI (any of criteria 1, 2, or 3), for a cumulative total at 5 years of 82/10 879 (0.8%) patients. Among the 82 patients, by individual criteria, 9 patients met Criterion 1, 44 met Criterion 2, and 43 met Criterion 3 (some patients met multiple criteria).

Most patients with possible severe DILI events were women (52/82; 63.4%) and aged ≤55 years (61/82; 74.4%), with a mean age of 47 years at REMS enrollment (Table 2). Data on racial/ethnic background and alcohol consumption were unavailable for >50% of patients in this group. No obvious associations of patient baseline characteristics with possible severe DILI events were evident. No definitive inferences can be drawn regarding the impact of medical history, concurrent conditions, or concomitant medication on risk of possible severe DILI, given small numbers that prevent valid comparisons.

**Figure 2:**
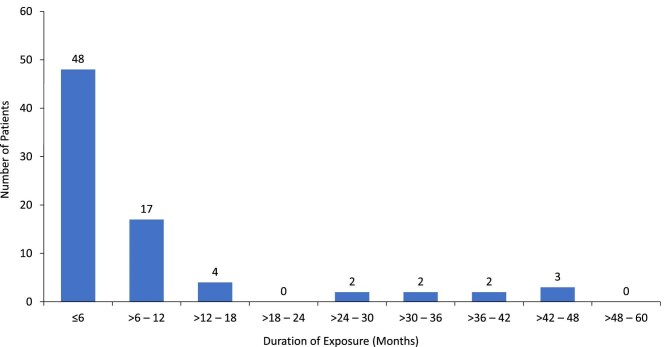
Number of patients with possible severe DILI (*n* = 78) by duration of tolvaptan exposure. The total patient number excludes four patients for whom time to event could not be calculated.

Time to event data were calculated excluding four patients for whom time to event could not be determined. For the resulting 78-patient analysis set, the median time to the first event of possible severe DILI was 138 days, interquartile range of 85 to 293 days. Distribution of the incidence of possible severe DILI by duration of tolvaptan exposure suggests greatest risk in the first 12 months of treatment (Fig. 2).

**Figure 3:**
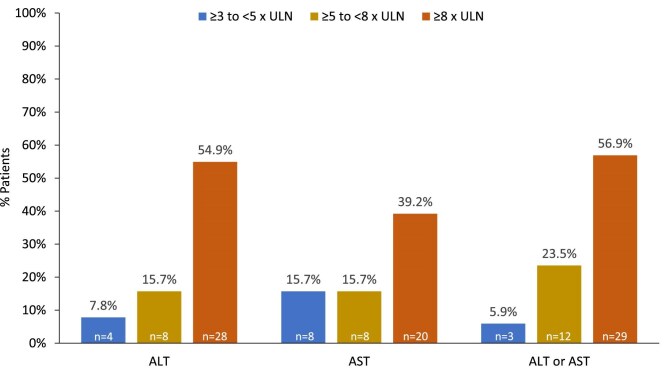
Highest ALT and/or AST elevations in patients with possible severe DILI for whom laboratory data on transaminase abnormalities were available (*n* = 51). The figure excludes patients with possible severe DILI for whom the relevant laboratory data were not provided.

Laboratory data on transaminase abnormalities were available for 51/82 of the individuals with possible severe DILI. The highest ALT and/or AST elevations (≥3, ≥5, or ≥8× ULN) occurring in these individuals are shown in Fig. 3. One patient met Hy's Law criteria, as reported in the 3-year interim analysis, with liver enzymes returning to normal after tolvaptan discontinuation [10]. No new Hy's Law cases have been confirmed since that time.

Of the 82 cases of possible severe DILI, 40 were initially reported as serious and potentially fatal. Four of the 40 patients were confirmed by Otsuka GPV to have developed events suggestive of serious and potentially fatal liver injury. All four confirmed cases, which were published with the 3-year interim data, recovered after tolvaptan was withdrawn [10]. No new cases have been confirmed since that time. It should be noted that confirmation requires sufficient information for complete and adequate medical assessment, and that many of the reported cases were lacking the necessary data. In most of the reported cases, a pharmacist or nurse was the reporter and not the prescribing physician. Missing data issues included not reporting any laboratory results, lack of evidence to support the presence of severe DILI (e.g. only minor laboratory elevations, absence of symptoms, no need for treatment or intervention), and absence of relevant medical history and clinical details. Follow-up efforts to obtain missing information are ongoing.

### Outcomes in patients with possible severe DILI

Outcomes data for the 82 patients with possible severe DILI are limited to information available during the 5-year reporting period. Outcomes data were not reported for 33/82 (40%) of patients, and multiple follow-up attempts have been made or are ongoing to obtain the missing information for each patient. For those with known outcomes, 26/82 (32%) had recovered/resolved at the end of the reporting period, 9/82 (11%) were recovering/resolving, 13/82 (16%) had not recovered, and in 1/82 (1%) the outcome was fatal. In the last event, the cause of death was not considered to be related to the possible severe DILI. The patient had *Clostridium difficile* infection and lactic acidosis leading to septic shock. Hepatic steatosis developed as a complication of sepsis, which led to a designation of possible severe DILI. The cause of death was determined to be *C. difficile* infection leading to septic shock. As previously reported, an additional patient with possible severe DILI died from a post-procedural hemorrhage and shock. The death occurred after tolvaptan had been discontinued for transaminase elevations and the outcome of the elevations was reported as recovering/resolving. Accordingly, this death was also considered to be unrelated to tolvaptan [10].

### Hospitalizations and liver transplant

Among patients with possible severe DILI during the 5-year follow-up period, 11 were hospitalized and three underwent liver transplant. The liver transplants were not reported as life-threatening events and were assessed as unrelated to tolvaptan by the GPV team. The first patient had underlying polycystic disease and was on a liver transplant list, as previously reported [10]. The second patient had no information provided regarding the reason for transplant, after which tolvaptan was restarted. The third patient's medical history included polycystic liver disease and hepatomegaly, but only limited information was provided regarding the time of and reason for transplant. The transplant took place ∼4 years after tolvaptan initiation. Given that there were no REMS patients with a fatal outcome attributed to tolvaptan or with liver transplant required due to DILI, no instances of confirmed severe DILI by FDA criteria have been identified.

### Comparison with clinical trials in ADPKD

The incidence proportion of possible severe DILI by all three criteria on a population basis was 0.8% in the REMS (82/10 879 patients) and 5.5% (151/2743 patients) in the clinical trial program (TEMPO 3:4, REPRISE, and their extension trials). The incidence rate per 100 patient years was 0.52 (82 events/15 817 patient years) in the REMS, which was significantly lower (IRR, 0.329; 95% CI, 0.252 to 0.431, *P* < .0001) than 1.57 (154 events/9786 patient years) in the clinical trials.

Comparison of incidence rates for possible severe DILI by individual qualifying criteria indicated that the difference between the REMS and clinical trials was largely driven by Criterion 3. The IRR for the REMS versus clinical trials was 0.506 (95% CI, 0.210 to 1.222; *P* = .1226) for Criterion 1 and could not be calculated for Criterion 2 because no cases met Criterion 2 in the clinical trials. The IRR (REMS versus clinical trials) for Criterion 3 was 0.186 (95% CI, 0.132 to 0.262; *P* < .0001).

## DISCUSSION

A similar proportion of patients was identified with possible severe DILI in these 5-year data as in earlier REMS reporting periods. At the 3-year interim analysis, 60/6711 (0.9%) of tolvaptan-naïve US REMS patients met criteria for possible severe DILI, and 69/8764 (0.8%) met these criteria in the 4-year interim analysis [10, 11]. At the time of this 5-year analysis and using the same criteria, 82/10 879 (0.8%) of patients were identified as having possible severe DILI.

The FDA requires comparison of the risk of severe DILI in the post-marketing setting with that of the tolvaptan clinical trials [7]. The incidence of possible severe DILI was significantly lower in the REMS at 5 years (0.52 events per 100 patient years) than in clinical trials (1.57 events per 100 patient years). This difference may be in part because liver function testing in the post-marketing setting is per label once monthly for the first 18 months of treatment, whereas the TEMPO 3:4 trial and part of the TEMPO 4:4 trial were conducted with longer intervals between liver function tests. Liver function abnormalities may have taken longer to detect and manage before the current testing schedule was mandated, which validates the recommendation of monthly testing in the real world. On the other hand, only 35% of the REMS patients were exposed to tolvaptan for longer than 18 months, the period of greatest susceptibility to tolvaptan-related liver adverse events, but the average patient exposure during the clinical trials was 3.57 years. Given this limitation of the present analysis, the comparison between the 5-year REMS data and the clinical trial data should be interpreted with caution. Despite this limitation, the data available so far support earlier observations from clinical trials that the greatest risk of DILI is in the first 18 months of treatment, with a lower incidence thereafter [2, 3].

Interestingly, patients who took tolvaptan for longer durations had higher average daily doses than those who took tolvaptan for shorter durations, with the reasons for the trend unclear.

No specific risk factors for tolvaptan-related DILI have been identified from patient demographics, medical history, or concomitant medications. One new fatality in a patient with possible severe DILI has been reported since the previous REMS reporting period, however, this fatality was deemed to be unrelated to tolvaptan because the hepatic event was a complication of sepsis that developed in a patient with *C. difficile* infection and lactic acidosis.

Another important limitation to this study is that the confirmation of serious and potentially life-threatening DILI events requires sufficient data for adequate assessment. Detailed and accurate data collection during post-marketing pharmacovigilance has been more challenging than within the more structured environment of clinical trials. This lack of sufficient data for cases of possible severe DILI in the REMS limited the reporting. The incidence of confirmed serious and potentially life-threatening DILI events reported here could change as follow-up attempts generate more detailed data. Nonetheless, despite the limitations of this study, data from the US REMS support that adherence to the per-label liver function testing schedule enables prompt detection and appropriate action and reduces the risk of severe outcomes in real world clinical practice.

## Data Availability

To submit inquiries related to Otsuka clinical research, or to request access to individual participant data (IPD) associated with any Otsuka clinical trial, please visit https://clinical-trials.otsuka.com/. For all approved IPD access requests, Otsuka will share anonymized IPD on a remotely accessible data sharing platform.
